# Analysis of *Phaseolus vulgaris* Response to Its Association with *Trichoderma harzianum* (ALL-42) in the Presence or Absence of the Phytopathogenic Fungi *Rhizoctonia solani* and *Fusarium solani*

**DOI:** 10.1371/journal.pone.0098234

**Published:** 2014-05-30

**Authors:** Jackeline L. Pereira, Rayner M. L. Queiroz, Sébastien O. Charneau, Carlos R. Felix, Carlos A. O. Ricart, Francilene Lopes da Silva, Andrei Stecca Steindorff, Cirano J. Ulhoa, Eliane F. Noronha

**Affiliations:** 1 Department of Cellular Biology, University of Brasilia (UNB), Brasilia, Distrito Federal, Brazil; 2 Biological Sciences Institute, Federal University of Goiás (UFG), Goiânia, Goiás, Brazil; University of Nebraska-Lincoln, United States of America

## Abstract

The present study was carried out to evaluate the ability of *Trichoderma harzianum* (ALL 42-isolated from Brazilian Cerrado soil) to promote common bean growth and to modulate its metabolism and defense response in the presence or absence of the phytopathogenic fungi *Rhizoctonia solani* and *Fusarium solani* using a proteomic approach. *T. harzianum* was able to promote common bean plants growth as shown by the increase in root/foliar areas and by size in comparison to plants grown in its absence. The interaction was shown to modulate the expression of defense-related genes (Glu1, pod3 and lox1) in roots of *P. vulgaris*. Proteomic maps constructed using roots and leaves of plants challenged or unchallenged by *T. harzianum* and phytopathogenic fungi showed differences. Reference gels presented differences in spot distribution (absence/presence) and relative volumes of common spots (up or down-regulation). Differential spots were identified by peptide fingerprinting MALDI-TOF mass spectrometry. A total of 48 identified spots (19 for leaves and 29 for roots) were grouped into protein functional classes. For leaves, 33%, 22% and 11% of the identified proteins were categorized as pertaining to the groups: metabolism, defense response and oxidative stress response, respectively. For roots, 17.2%, 24.1% and 10.3% of the identified proteins were categorized as pertaining to the groups: metabolism, defense response and oxidative stress response, respectively.

## Introduction

*Phaseolus vulgaris* L., the common bean, is one of the most ancient cultivated crops in the world. It is of social, economic and dietary importance in many countries, principally in South America, Africa and Asia [1]. The dry beans are an important source of dietary proteins and carbohydrates, and also contain important vitamins, minerals, fibers and phenol compounds with antioxidant properties.

The Brazilian Ministry of Agriculture (CONAB) estimated for 2010/11 a dry bean production of 3.45 million tons, with each hectare of planted soil yielding 965 tons. These values are 6% percent greater than those obtained for the previous five years. Most of this production is used to attend to the domestic market, due to increased internal demand and losses caused by fungal diseases [2]. Fungal diseases impact Brazilian and worldwide dry bean production with losses of up to 60%, mainly caused by the fungi *Colletrotrichum lindemuthianum* (anthractonose), *Sclerotinia sclerotiorum* (white mold), *Rhizoctonia solani* (root rot), *Fusarium oxysporum* and *Fusarium solani* (fusarium wilt), and other soil-borne fungi [3]. The pathogenic fungal hyphae penetrate the plant directly or through wounds and natural openings, promoting root rot, reducing seedling vigor and consequently causing reduction in crop production.

The losses of crops produced in Brazilian soils are remarkable and are caused mainly by *F. solani*, *R. solani* and *S. sclerotiorum* [3]. These fungi can be spread over long distances by wind, water and agricultural equipments, through particles of soil, infected crop residues and contaminated seeds [3], [4]. Chemical treatment using fungicides is the most common worldwide strategy to control these diseases, but the chemicals used for this purpose have a limited action against these phytopathogenic fungi. In addition, there are environmental and human health disadvantages related to chemical use [5].

*Trichoderma* species, notably, *Trichoderma harzianum* have been commercially used as biological control agents against the phytopathogenic fungi *Botrytis cinerea*, *Phytophthora palmivora* and *Pythium* spp., in papaya, tobacco, castor beans and bean crops [6]–[8]. They are able to reduce plant colonization and host fungal growth using a combination of specific mechanisms such as mycoparasitism, antibiosis and competition for space and nutrients in the rhizosphere environment [9].

In addition to their mycoparasitic activity, *Trichoderma* species are rhizosphere-competent, being able to colonize and grow in association with plant shoots and roots as previously described for cucumber, cotton, maize, tomato, pepper, lettuce, bean and other plants [10]–[12]. This association changes the plant development (growth and metabolism) and also induces defense responses and resistance to subsequent fungal attack, as previously described for the associations *T. harzianum*/*Brassica napus*, *T. harzianum* T22/*Zea mays, T. harzianum* T39/grapevine and *T. harzianum/Brassica oleraceae* [13]–[17].

The activation of plant defense mechanisms occurs via a succession of events and signals that start with the recognition of pathogenic/nonpathogenic micro-organisms by the plant and culminates in physical barrier formation and chemical response activation leading to protection against invasion. The plant defense response includes three main mechanisms: a) Hypersensitive Response (HR), b) Systemic Acquired Resistance (SAR), that includes induction of pathogenesis-related proteins (PR- proteins) and c) Induction of Systemic Resistance (ISR), which includes production of flag compounds, such as salicylic acid and hydrogen peroxide [18]. The mechanisms used by *Trichoderma* species to trigger the plant defense response are not completely understood, however studies concerning differential gene expression and alterations in the protein expression patterns of leaves and roots of host plants during association with *Trichoderma* species have been carried out [5], [18], [9]. Nevertheless, studies describing the interaction between common bean plants and *T. harzianum* as well as common bean with *F. solani* have not been published.

Our research group has previously worked with the isolation, identification and characterization of biological control activity of *Trichoderma* strains isolated from Brazilian Cerrado soil in order to identify agents exhibiting efficient biological control against the fungi *R. solani*, *F. solani*, *S. sclerotiorum* and other pathogens that impact dry bean crop production in Brazil. Our results so far have suggested that the *T. harzianum* Rifai (anamorph) ALL42 isolate has great potential for inhibiting the growth of *F. solani* and *R. solani in vitro* [19], [20]. This isolate also reduces fungal disease incidence in common bean, and shows the capacity to facilitate an increase in plant growth. In order to study the mechanisms by which *T. harzianum* ALL-42 interacts with and changes common bean metabolism, we analyzed its ability to induce the host defense response. In addition we used a proteomic approach to map proteins produced by plants cultivated in the presence or absence of the phytopathogenic fungi *R. solani* or *F. solani* together with this mycoparasitic fungus. The differentially expressed proteins were identified by peptide mass fingerprinting using a MALDI-TOF/TOF mass spectrometer. The mapping of proteins with crucial roles in the induction of defense response and the description of the main mechanisms involved in this interaction can contribute to the development of rational strategies for managing fungal diseases in Brazilian crop production.

## Materials and Methods

### Soil Preparation

*P. vulgaris* seeds were germinated and cultured in the presence or absence of the phypathogenic fungi and *T. harzianum* ALL 42 isolate, obtaining four main conditions termed: TT (untreated seeds); 42T (seeds treated with ALL 42 spores); and double fungal treatments: 42 FS and 42 RS (seeds treated with ALL 42 spores and with *F. solani* or *R. solani*). The cultures were carried out using the sterile substrate Plantmax previously autoclaved for 30 min at 120°C.

For the experiments using the double fungal treatment (*T. harzianum*/phytopathogenic fungi) the soils samples were previously infected with the phytopathogenic fungi [21]. Sorghum seeds, humidified with distilled water and autoclaved, were used as substrate for the phytopathogenic fungal growth. Briefly, 5 culture disks of *R. solani* or *F. solani*, previously cultured in MYG solid medium (per liter: 5 g of malt extract, 2.5 g of yeast extract, 10 g of glucose and 10 g of agar), were inoculated into the sorghum media and incubated at 28°C for 10 days, until complete colonization. The aggregate-colonized sorghum seeds were manually disaggregated and dried at room temperature for three days. Finally, the colonized sorghum was triturated, sifted (20 mesh) and used for soil infection, with 0.5 g of *R. solani*-colonized sorghum and 0.8 g of *F. solani*-colonized sorghum per 1.4 kg of sterile soil.

### Fungal Growth Conditions

*T. harzianum* ALL 42, isolated from cerrado soil and previously characterized as an effective biocontrol agent [19], as well as the phytopathogens *F. solani* and *R. solani* were obtained from the Embrapa (CNPAF, Goiânia, Brazil) research center’s culture collection and maintained in MYG culture medium at 4°C with periodic subcultures.

Conidia from *T. harzianum* ALL 42 were obtained as previously described [21]. Briefly, distilled water (25 mL) was added to parboiled rice (100 g) in 500 mL conical flasks, autoclaved for 20 min and used as substrate for fungal growth. After cooling, 3 disks of *T. harzianum* ALL 42 mycelia were added to the flasks containing rice and incubated at 28°C for 7 days with a photoperiod of 12 hours. This culture was then washed with 100 mL of sterile distilled water under stirring to obtain the spores further used to inoculate the soil in co-cultivation growth bioassays.

### Co-cultivation Growth

*P. vulgaris* growth was carried out in triplicate using 10 plants for each experimental replicate (experiment 1, 2 and 3), under four main conditions (TT, 42T, 42FS and 42RS), totaling 120 plants. Seeds of *P. vulgaris* were washed with 1% hypochlorite solution for 1 min and then with sterile distilled water [6]. Rinsed seeds were immersed in a *T. harzianum* spore suspension containing 2.4×10^8^ spores per mL and sown in 500 mL cups containing sterile soil, for the 42T condition. Control plants (TT) were cultivated with rinsed seeds sown in sterile soil. For the two other conditions, *P. vulgaris* seeds were sown in infected soil, prepared as described above.

The plants corresponding to each growth condition and experiment were collected after 21 days and thoroughly rinsed with sterile distilled water until complete removal of soil substrate and superficial fungal hyphae. The roots and leaves (an average of 5 for each plant) of ten plants from the experiment 1 were pooled and immediately stored at −80°C. This procedure was also carried out for replicates in experiments 2 and 3. These tissues were used in the mapping of expressed proteins using 2DE electrophoresis. Three replicate gels were prepared for each experiment (experiments 1, 2 and 3) totaling 9 gels for each treatment (TT, 42T, 42FS and 42RS).

The leaf area, plant size and root volumes were measured for plants grown in the presence (42T n = 30) or absence (TT n = 30) of the *Trichoderma* isolate. A total of 10 plants were randomly chosen for leaf collection. These tissues were digitalized and the biometric parameters were determined using the software packages Image J 1.47 (free download: http://rsbweb.nih.gov/ij/index.html) and WhinRHIZO^©^. Statistical analyses were performed using student’s *t*-test with the software R (*p*<0.05).

### Expression Analysis of Common Bean Defense-Related Genes

Total plant RNA was extracted using the Invisorb RNA kit II, according to the manufacture’s manual and treated with DNase I (Invitrogen). Treated RNA (5 µg) was reverse transcribed using the Revertaid First Strand cDNA synthesis kit (Fermentas). Each reaction, with a volume of 20 µl, contained 10 µl of MAXIMA SYBR-green PCR Master mix (Fermentas), forward and reverse primers (500 nM each), cDNA template, and nuclease free water.

PCR cycling was performed using the iQ5 real-time PCR system (Bio-Rad) with a standard protocol: 10 min at 95°C (1 cycle), 15 s at 95°C followed by 1 min at 60°C (40 cycles), a melting curve of one min at 95°C followed by 30 s at 55°C and a final ramp to 95°C with continuous data collection (1 cycle) to test for primer dimers and nonspecific amplification. The β-actin (EU581898) transcript was used as internal reference to normalize the amount of total RNA present in each reaction. Gene expression levels were calculated from the threshold cycle according to the 2^−ΔΔCT^ method (Livak and Schmittgen, 2001). The experiment was carried in triplicate for each sample and results were compared by one-way ANOVA with Dunnett’s post test (α = 5%) to analyze the differences between conditions related and controls using GraphPad Prism version 5.00 for Windows.

First we analyzed the expression pattern of plant defense-related genes to check root colonization. In this case, four genes were analyzed: chit (AY357300.2, which encodes a chitinase), glu1 (DQ093563.1, which encodes a β-1,3-glucanase), pod3 (AF 485265.1, which encodes a peroxidase) and lox1 (U76687.2, which encodes a lypoxygenase). To validate differential protein accumulation 5 genes corresponding to spots R10, R19, R26, R31, R40 (chalcone isomerase, acyl-coA-binding protein, NAC1 domain protein, glyceraldehyde-3-phosphate dehydrogenase and PR1-like protein, respectively) were chosen according to their presence or absence in the proteomic maps (Tables 1 and 2). Primers sequences were designed based on sequences available in the GenBank database. Root of plants unchallenged and challenged for 7, 14 and 21 days by *T. harzianum* and phytopathogenic fungi were used to RNA extraction and qRT-PCR reactions, as above described.

**Table 1:** List of *P. vulgaris* leaf proteins, identified by MS analysis.

LEAF TISSUE	
			MASCOT			MW	p*I*						
Spot n°	Protein	Acession n° (Bean database and NCBI)	Seq. Cov. (%)	Score		^a^Exp/^b^Theo	^a^Exp/^b^Theo		Matched protein sequences (NCBI database)			presence/absence in control plants	
L3	*putative serine/threonine kinase*	1558202_2	29%	78		23,1/22,049	6,7/8,97		EFPLFNFSCISVATNNFSEENKLGKGGFGPVYKGKLPDGEQIAVKRLSRRSGQGLEEFKNEMMLIAKLQHRNLVRLMGCSIQGEEKLLVYEYMPNKSLDCFLFDPFKQTQLDWRRRFEIIEGIARGLLYLHRDSRLRIIHRDLKASNVLLDESMNPKISDFGLARIFGGNQNEANTNRVVGTYGYMAPEYAM			present	
L6	*Unknown*	66974_3	22%	65		17,3/20,164	8,5/9,46		ELTISFEGEVYVFPAVTPEKVQAVLLLLGAQEMTNSAPTSDILLQQNYQDIREINDPSRSSKLSRRFASLVRFREKRKERCFEKKIRYSCRKEVAQRMHRKNGQFASMKEDYKSPAENWDSSNGTPCPDSTERRCQHCGISEKSTPAMRRGPAGPRSLCNACGLMWANKGTLRDLSK			present	
L7	*Ribulose bisphosphate carboxylase Small subunit*	gI809069	48%	76		15.7/16	8.6/8.99		TSVANNGGRVQCIQVWPTVGKKKFETLSYLPPLTKQQLAKEVDYLLRKGWVPCLEFELEHGFVYREHNKSPGYYDGRYWTMWKLPMFGCTSSQVLKELYEAQTAHPDGF IRIIGFDNVRQVQCISFIAYKPPGY			present	
L8	*Unknown*	48950_3	34%	68		24,54/23,036	9,4/9,94		MGNALGGKKTTKVMKIDGETFKLKTPIKVCDVLKDHPGLVLLESEAVKHYGIRAKPLEAHKELMPKRLYFLVELPKEVTVAPRRVRSGINMSAKERPREPRFGVAGRASDLTDHGNPRRAKKRLLESGGGVRLKMRLPKAEVERLMRGCETEAEAAEKIMGLCMANNGGGVEARNGDGEVKGRVGESTKAREKRVSFMPINEGGSPIAVAS			present	
L9	*SGF14* *h*	1716582_3	18%	64		27,9/29,152	9,66/5,27		MAAAPTPREKNVYMAKLAEQAERYKKMVEFMEKVSAAANNEELTVEERNLLSVAYKNVIGARRASRRIISSIERKEESRGNEDHVAVIRDYRSKIESELSNICDGILKLLDSRLIPSASSGDSKVFYLKMKGDYHRYLAEFKTGAKRKEAAESTLAAYKSAQDIANAELPPTHPIRLGLALNFSVFYYEILNSPDRACNLAKQAFDEAIAELDTLGEESYKDSTLIMQLLRDNLTLWTSDMQDDGADEIKEAAPKQDDQ			present	
L10	*conserved hypothetical protein*	3177_3	30%	71		17/17,493	3,5/4,66		SLHAFHHAPPHNVRGSVWCVCRSKESDSEGSPAEGDAKSQELLAQIAMLQTQKVRLTDFLDERSAYLSQFGEEAKAEFDKIGEDALKELDEAGARITANIESEMLAFEESSELNRVEIEESEKKIEEFEGQMEKNRNEGLFFKNLGEKALDDKEK			present	
L15	*unknown*	54846_3	33%	107		21,3/25,81	9,5/9,76		TYVIKIPKDQVYRVPPPENARRYDQYARRKHRRSRCCCCFCWLIGILFILVVLLAIAAGVLYLVFRPEAPKYSIENITVRGINLTSPSSVAAISPEFNVTVKADNPNDKIGIRYLKDSSAEVFYKDARLCNGALPAFYHPSNNVTVFGTALRGDGIELRSEDRRALLEAQTKRRVPLTVRIRAPVKIKVGSIRTWKITVKVNCDVTVNELTAQAKIVSKRCSYDVDLW			present	
L17	*unknown*	70740_3	37%	67		16,9/16,178	10.13/11,33		KPISSPGRTEKFPPPLMRFLRNNASSRSRGRSRTTTAMFLRKKNTNNIETQEPSSPKVTCMGQVRVKRSASKRVPSAGAGTPTKFRCCSWVPHALFFHRLIKPEVCFPFQCKQVWPNWXFLQRKKRDSKVTETSSPKTELN			absent	
L20	*unknown*	92785_2	10%	61		34/36,75	5,2/6,12		MSDERAVHPDCRNASNPYHECSDYCFRVIAEAKIRSQQQESEVGQASGGNNSKQAIPDESYVEKEIHNGRPDLEENSDSDPDQPAVQEAEQEVDYTKLSARQKKWMELRSKMQEAKKRNQIEIAAEKKRMEAPTESRGVSKQKWLEDRKNKIGKLLDANGLDMTKAYMLDTQEAAEEKYKKWEKDPAPFGWDVFNQKTLYNAYKKRTKNVEVDVEEYNRMKEADPEFYRDASSLQYGKAPKISEEKIDRMVRELKDRDEKRNSFSRRRRFHEEKDIDSINDRNEHFNKKIERAFGKYTLEIKNNLERGTALPD			present	
L22	*rubisco activase*	455_1	21%	102		48,2/48,23	6,8/6,49		MAASLSTVGAVNRTLLNLNGSGGGASGPSSAFFGTSLKKVISSRVPNSKLTSGSFKIVAADKEIEETQQTEGDRWRGLAYDVSDDQQDITRGKGLVDSLFQAPMDAGTHYAVMSSHEYLSAGLRQYDFDNMKDGFYIAPAFLDKLVVHIAKNFMTLPNIKVPLILGVWGGKGQGKSFQCELVFAKMGINPIMMSAGELESGNAGEPAKLIRQRYREASDLIKKGKMCVLFINDLDAGAGRLGGTTQYTVNNQMVNATLMNIADNPTNVQLPGMYNKEDNARVPIIVTGNDFSTLYAPLIRDGRMEKFYWAPTREDRIGVCKGIFRTDGVPEKDIVELVDKHPGQSIDFFGALRARVYDDEVRKWISGVGVDSVGKKLVNSKEGPPTFDQPKMTLDKLLLYASMLVQEQENVKRVQLADQYLNEAALGNANEDAIKSGSFFK			absent	
L23	*carbonic anhydrase*	749_3	25%	81		29,5/35,6	7,6/8,09		MSTSSINGWCLSSISPAKTSLKKATLRPSVFATITTPSSPSSSSSSSFPSLIQDRPVFAAPSPIITPTVRGDMAKEYEQAIEELQKLLREKSELKATAAEKVEQITASLGTTSSDGIPSSEASERIKTGFLYFKKEKYDKNPALYGELAKGQSPKFMVFACSDSRVCPSHVLDFQPGEAFVVRNVANIVPPYDQSKYSGTGAAIEYAVLHLKVSNIVVIGHSACGGIKGLLSFPYDGTYSTDFIEEWVKIGLPAKAKVKTQHGDAPFGELCTHCEKEAVNVSLGNLLTYPFVRDGLVNKTLALKGGYYDFVKGSFELWGLNFGLASSFSV			present	
L25	*glutathione S-transferase GST 13*	40460_3	15%	66		30/25,19	5,4/5,53		MASYHEEEVRLLGKWASPFSNRVDLALKLKGVPYKYSEEDLANKSADLLKYNPVHKKVPVLVHNGNPLPESLIIVEYIDETWKNNPLLPQDPYERALARFWSKTLDDKILPAIWNACWSDENGREKAVEEALEALKILQEALKDKKFFGGESIGLVDIAANFIGYWVAILQEIAGLELLTIEKFPKLYKWSQEFINHPVIKEGLPPRDELFAFFQASAKK			absent	
L26	*cinnamoyl-CoA reductase family*	Pa_04ANAA3_T7_068_B05_26FEB20	27%	65		16,8/17,71	5,8/5,61		VNVLTAAKEAGVRRVVVTSSVSAIIPSPNWPGDVPKREDCWADVEFCKQKGLWYSLSKTLAEKAAWDFAKESGLDVVVVNSGTVMGPIITPRLNASMLMLLRLLQGSDETYEDIFMGSVHLNDVTLAHILVYENKSAAGRHLCVESISRFGDFAAKVAELYP			absent	
L27	*uncoupling protein 1a*	18378376	30%	80		26/25,9	9,5/9,63		LQLQKQAATGDVVSLPKYKGMLGTVATIAREEGLSALWKGIVPGLHRQCLYGGLRIGLYDPVKTFYVGKDHVGDVPLSKKILAAFTTGAFAIAVANPTDLVKVRLQAEGKLPPGVPRRYSGSLNAYSTIVRQEGVGALWTGLGPNIARNGIINAAELASYDQVKQTILKIPGFTDNVVTHLLAGLGAGFFAVCIGSPVDVVKSRMMGDSSYRNTLDCFIKTLKNDGPLAFYKGFLPNFGRL			present	
L35	*Aminomethyltransferase*	3140_3	39%	116		44,7/44,205	8,3/8,78		MRGGLWQLGQLVTRRLAHGDKKAVARRCFASEAELKKTVFHDFHVVHGGKMVPFAGWSMPIQYKDSIMDSTINCRQNGSLFDVSHMCGLSLKGKDAAPFLEKLVIADVAGLAPGTGTLTVFTNEKGGAIDDSVITKVTDDHIYLVVNAGCRDKDLAHIEEHMKAFKAKGGDVSWHIHDERSLLALQGPLAAPVLQHLTKEDLSKLYFGEFRVLDINGSQCFLTRTGYTGEDGFEISVPSEHGVDLAKAILEKSEGKIKLTGLGARDSLRLEAGLCLYGNDMEQHITPIEAGLTWAIGKRRRAEGGFLGADVILKQLEEGPSIRRVGFISSGPPPRSHSEIQDEGGKNIGEITSGGFSPCLKKNIAMGYVKSGLHKAGTKVKIIIRGKANEGVVTKMPFVPTKYYKPS			absent	
L38	*GTP-binding protein*	60773_1	46%	69		17,5/9,67	9,8/9,92		MATVMQKIKDIEDEMARTQKNKATAHHLGLLKAKLAKLRRELLTPSSKGAGGAGEGFDVTKSGDSRVGLVGFPSVGKSTLLNKLTGTFSEV			present	
L41	*ribulose-1,5-bisphosphate carboxylase/oxygenase large subunit*	44810_2	15%	67		58/52,62	4,9/6,09		MSPQTETKASVGFKAGVKDYKLTYYTPDYETKDTDILAAFRVTPQPGVPPEEAGAAVAAESSTGTWTTVWTDGLTSLDRYKGRCYGLEPVAGEENQYIAYVAYPLDLFEEGSVTNMFTSIVGNVFGFKALRALRLEDLRIPTSYIKTFQGPPHGIQVERDKLNKYGRPLLGCTIKPKLGLSAKNYGRAVYECLRGGLDFTKDDENVNSQPFMRRDRFLFCAEAIFKSQAETGEIKGHYLNATAGTCEEMMKRAVFARELGVPIVMHDYLTGGFTANTSLAHYCRDNGLLLHIHRAMHAVIDRQKNHGMHFRVLAKALRLSGGDHVHAGTVVGKLEGEREITLGFVDLLRDDFVEKDRSRGIYFTQDWVSLPGVLPVASGGIHVWHMPALTEIFGDDSVLQFGGGTLGHPWGNAPGAVANRVALEACVQARNEGRDLAREGNEIIREASKWSPELAAACEVWKEIKFEFQAMDTL			present	
L42	*Full = Ribulose bisphosphate carboxylase/oxygenase Phaseolus vulgaris*	gI10720248	25%	83		26/42,7	6,1/8,7		MAASLSTVGAVNRTLLNLNGSGGGASGPSSAFFGTSLKKVISSRVPNSKLTSGSFKIVAADKEIEETQQTEGDRWRGLAYDVSDDQQDITRGKGLVDSLFQAPMDAGTHYAVISSHKYLSAGLRQYNFDNIKDGFYIAPAFLDKLVVHIAKNFMTLPNIKVPLILGVWGGKGQGKSFQCELVFAKMGINPIMMSAGELESGNAGEPAKLIRQRYREASDLIKKGKMCVLFINDLDAGAGRFSTLYAPLIRDGRMEKFYWAPTREDRIGVCKGIFRTDGVPEKDIVELVDKHPGQSIDFFGLRARVYDDEVRKWISGVGVDSVGKKLVNSKEGPPTFDQPKMTLDKLLLYASMLVQEQENVKRVQLADQYLNEAALGNANEDAIKSGSFFK			present	

**Table 2:** List of *P. vulgaris* root proteins, identified by MS analysis.

ROOT TISSUE	
			MASCOT		MW	p*I*		
Spot n°	Protein	Accession No. (Blast NCBI)	Seq. cov (%)	Score	Exp/(Theo)	Exp/(Theo)	Matched protein sequences in bean database/NCBI	Control plants protein spot presence/absence
R3	*conserved hypothetical protein*	1563790_2	36%	72	13/10,9	5.39/9,83	DTKVALAFGMVAARRYGTDITLWHGLQGKGDPYRTLLREGITALLNSYNSLQFSYHPIGVVEHMNLALMGSNRSVLLTALRFKRANSGAGNVTCKFTTC	present
R4	*t-snare*	1070170_3	65%	66	4,1/4,75	8.13/9,056	MASSFDRWEKDPFFNAAEEVQESADRMESTYRTWIHSMRDASSPWNCDELRRDLQTTLGTAKWQLEEFERAARSSY	present
R5	*ripening related protein*	2678_1	17%	64	17/17,767	6.30/5,48	MVLLGKISTEIGVHATAEKWFNLFAKQLHDVQHLAERVHGTKLHQGEDWHHNDTIKQWTYVIDGKVTTCHESIESVDEENNTIYYKLFGEDIDHRFKVFKLIFQAIDKENHGVIIKWTIEYEKLDGEVEPPYGYIEYLHKCTSDIDANLLKA	absent
R6	*peptide deformylase*	1006202_1	48%	65	18/10,21	6.14/5,25	SSQTCSARAGWFLGLGADSKKTNLPDTVKAGDPVLHEPAQDVDPNEIKSERVQKIIDDMIQVMRKAPGVGLAAPQIGIPLRIIVLEDTKEYISY	present
R7	*ACBP3 (ACYL-COA-BINDING DOMAIN 3); acyl-CoA binding*	1559111_1	27%	77	26/22,193	6.93/4,27	AAASSDIERQIEESMVEPVFPSESTVLSPVQAATCVGSELKVEEVVMEVGSNVVLESPLKSRSDIAVKEEIAEANEGETREFDEKRDVESVEDSCTEIEVSTVENGVKENYYDDEDDDWEGIERSELEKEFMAATEFVTHECDRLESVGSNVKMELYGLHKVATEGPCREPQPMPLKLSARAKWNAWQKLGNMNPEVAM	present
R8	*putative glutathione S-transferase [Phaseolus acutifolius]*	gi|21217741	26%	72	26/25	6.77/5.57	MVLKVYGPTCASTKRVLVCLLEKEVEFEVIPVDLTKGEHKDPEFLKLQPFGVVPVIQDGDYTLYESRAIMRFYADKFRSQGVELLGRTAEERGVVEQWLEVEAHNFHPPAYDLAVHVLFASLFGITPDPKVIEESEAKLLKVLDVYEDRLSKGKYLGGDFLSLADISHLPFIDYIVNKMNKGYLIKERKHVSDWWDDISSRPSWKKVNQLYPPPV	present
R10	*chalcone isomerase (EC 5.5.1.6) - kidney bean (fragment)*	4178_2	49%	105	26,5/15,28	5.38/5,63	LDFYRDIISGPFEKLIRGSKILQLSGTEYSRKVMENCVAHLKSVGTYGDAEAKGIEEFAEAFKKVNFPPGASVFYRQSPHGILGLSFSEDATIPGEEAVVIENKAVSAAVLETMIGEHAVSPDLKRSLASRLPAVLNGGIIV	present
R11	*Pto-like kinase SG5–3b [Phaseolus vulgaris]*	gi|14010525	39%	92	30,6/34	6.87/5.35	PGSGQGLPEFQTEIMVLSKIRHRHLVSLTGYCDERLEMILVYEYMEKGTLRDHLYNTKFPTLSWKARLQICIDSARGLHYLHKGAAGGIIHRDVKSTNILLDENHVAKVADFGLSRSGPLGTESYVTTGVKGTFGYLDPEYFRSQQLTEKSDVYSFGVVLWQVLCARAAIDPSLPRDQINLVWWGLLCKNKGTLQEIIDPSIKDQIDQNSLRKFSETIEKCLQEDGSDRPTMGDVLWDLEYAVQLQRGANAIQREPYEGSSSSVSASFQLPNVRRLPSLSTLSEADDTIVRNDESDSAVDYVFSQLKIDDAR	present
R12	*isoflavone reductase*	2132_1	28%	75	33/29,31	7.31/6,6	MAEKSKILIIGGTGYIGKHIVEASAKSGHPTFALVRESTVSDPAKAQLIDHFKALGVNLVHGDLYDHETLVKAIKQVDVVISTVGHLQLADQVKIIAAIKEAGNVKRFFPSEFGNDVDRVHAVEPAKSAFAIKVQIRRSIEEEGIPYTYVSSNYFAGYFLPTLAQPGVFAPPPPKDKVVIFGDGNPKAIFNKEEDIGTYTIRAVDDPRTLNKILYLRPPQNTYSFNELVALWEKKIGKTLEKIYVPEEKLLKDIEEAPIPINVIL	present
R15	*protein phosphatase 2C-like protein*	43064_3	14%	65	37, 43/31,08	7.21/6,36	MTGKDILHIMKVKAGFAPPDTGKGKGKISKHITHGFHLMKGKSAHPMEDYLVSEFKQEKDRELGLFAIFDGHLGHDVASYLQNHLFQNILKEHDFWTETESAVKRAYLETDEKILEQALVLGRGGSTAVTAILIDGQKLVVANVGDSRAVICENGKARQLSVDHEPSKEKKWIERRGGFVSKIPGDVPRVDGQLAVARAFGDRSLKMHLSSEPDVLVEEVDPHTEFLILASDGIWKVMSNEEAVESIRQIKDAQAAAKHLIEEAVSRKSKDDISCIVVRF	present
R16	*histone acetyltransferase complex component*	47122_2	41%	64	14,82/16,90	4,19/8,37	RLQELKEARAAGCRNSAEADRYLAQKRRREAEESGCRTKESAQGGPSNQGVPNALMSPDSAGKDLSGRPAGPATSSSVNEMDVTGYYGADLLSEPEKRLCCELRLPPAMYLKMQEQLSLQILAGTVAAKSDAHQLFKMDAMKIDRVYDMLIKKGI	absent
R17	*peroxiredoxin*	1721940_1	32%	69	19/17,33	5.86/5,41	MAPIAVGDAIPDGILAYLDDENKPQTVSIHSLAAGKKVIIFGVPGAFTPTCSLKHVPGFIERAEELKGKGVDEVICISVNDPFVMNSWAKTFPENKHVKFLADGAAKYTNALGLELDLTEKGLGVRSRRFALLVEDLKVKVANVESGGEFTVSSAEEIIKAL	present
R18	*unknown*	885_2	36%	65	19/24,4	5.71/8,83	QRSLIYAFVSRGTVILAEYTEFSGNFNTIAFQCLQKLPASNNKFTYNCDGHTFNYLVDNGFTYCVVADESIGRQVPVAFLERVKDDFLAKYGGGKAATAAANSLNKEFGSKLKEHMQYCVEHPEEISKLAKVKAQVSEVKGVMMENIEKVLDRGEKIELLVDKTENLHHQAQDFRNSGTKIRRKMWLQNMKVKLIVLAILIALILIIVLSVCRGFNC	present
R19	*acyl-CoA-binding protein*	1559111_1	27%	77	23/22,1	3.99/4,27	AAASSDIERQIEESMVEPVFPSESTVLSPVQAATCVGSELKVEEVVMEVGSNVVLESPLKSRSDIAVKEEIAEANEGETREFDEKRDVESVEDSCTEIEVSTVENGVKENYYDDEDDDWEGIERSELEKEFMAATEFVTHECDRLESVGSNVKMELYGLHKVATEGPCREPQPMPLKLSARAKWNAWQKLGNMNPEVAM	absent
R20	*14-3-3-like protein*	1091231_2	29%	80	29/10,82	4,35/5,39	SAPTTREDYEYMAKLADQSTRYEEMEEFMNKVYLSAXSYELTVEERNLLSVAYKNMIGARRASXRIISSIEQKEESRSNXDHCPVIHDYRSR	absent
R21	*14-3-3 protein*	282_1	36%	92	29/28,41	5.46/4,67	REEFVYMAKLAEQAERYEEMVEFMEKVSAAAENEELTVEERNLLSVAYKNVIGARRASWRIISSIEQKEESRGNEDHVSVIRDYRSKIESELSNICDGILKLLDSRLVPSAASGDSKVFYLKMKGDYHRYLAEFKTGGDRKEAAESTLSAYKSAQDIANSELPPTHPIRLGLALNFSVFYYEILNSPDRACSLAKQAFDEAIAELDTLGEESYKDSTLIMQLLRDNLTLWTSDMQDDGADEIKEAAPKADGE	present
R22	*glutathione S-transferase*	3838_2	28%	76	45/25,5	5.54/5,29	MASSQEEVTLLGATASPFVCRVKIALKLKGIEYKYVEENLGNKSEQLLKYNPVHKKVPVFVHGDKPLAESLVIVEYIDETWNNNPILPSDPYQRALARFWSKFIDDKIVGATWKSVFTADEKEREKNVAEASESLQFLENEIADKKFFGGEELGLVDIAAVYVAFWIPLVQEIGGLELLTSEKFPNLYKWSQEFVSHPIVKESLPPRDPVFGFFKGRYESLFASK	present
R24	*kinesin light chain, putative*	1563330_4	29%	62	15/16,22	8,5/10,6	IQGQGERKRAFLEVFXTKGVGGGSMFQKKKGAXMFEGGRGILNRSVGLFLKTFEGEGILQATYDVMGKSWKSEXDLRIFPXSXGRKSLEXQILILKNKKRGLVKLLKEAGKTRDRKAKSLENLIDPGSKRTKKEGTKRWPGLGFR	present
R26	*NAC domain protein NAC1*	60446_3	15%	83	35,2/34,34	6,5/8,66	MSNISMVEAKLPPGFRFHPRDEELVCDYLMKKVAHNDSLLMINVDLNKCEPWDIPETACVGGKEWYFYTQRDRKYATGLRTNRATASGYWKATGKDRSILRKGTLVGMRKTLVFYQGRAPKGKKTEWVMHEFRIEGPHGPPKISSSKEDWVLCRVFYKNREVSAKPRMGSCYEDTGSSSLPALMDSYISFDQTQTHADEFEQVPCFSIFSQNQTSPIFNHMATMEPKLPANHATNAYGGAPNLGYCLDPLSCDRKMLKAVLNQITKMERNPLNQSLKGSPSLGEGSSESYLSEVGMPHMWNNY	absent
R27	*fructokinase*	3813_1	34%	73	46/34,34	5,8/5,19	ATGTGLIASFGEMLIDFVPTVSGVSLAEAPGFLKAPGGAPANVAIAVARLGGKAAFVGKLGDDEFGHMLAGILKENGVRADGITFDQGARTALAFVTLRADGEREFMFYRNPSADMLLKPEELNLELIRSAKVFHYGSISLIVEPCRSAHLKAMEVAREAGCLLSYDPNLRLPLWPSADEARKQILSIWEKADLIKVSDVELEFLTGSDKIDDASALSLWHPNLKLLLVTLGEQGSRYYTKSFKGSVDAFHVNTVDTTGAGDSFVGALLSKIVDDQSILEDEPRLRDVLKFANACGAITTTQKGAIPALPKEEDALKIIK	present
R28	*monodehydroascorbate reductase*	5178_2	37%	94	49/24,6	6,2/5,03	AFYEGYYANKGVNIIKGTVAVGFTSNSDGEVNEVKLKDGRVLEADIVVVGVGGRPQTALFKGQVEEDKGGIKTDSFFKTNLSDVYAVGDVATFPLKLYGELRRVEHVDHSRKSAEQAVKAIKAAEDGKTIEEYDYLPYFYSRAFDLSWQFYGDNVGDTALFGDNEPASPKPKFGTYWIKDGKVVGVFLENGTPEENSAIAKVARVQPPVADVDQLAKEGLSFASKI	present
R30	*NAC domain protein NAC1 [Phaseolus vulgaris]*	gi|15148912	22%	73	36,3/34	8.5/8.32	MSNISMVEAKLPPGFRFHPRDEELVCDYLMKKLTHNDSLLMIDVDLNKCEPWDIPETACVGGKDWYFYTQRDRKYATGLRTNRATASGYWKATGKDRPILRKGTLVGMRKTLVFYQGRAPKGRKTEWVMHEFRIEGPHGPPKVSSSKEDWVLCRVFYKSREVSAKPSMGSCYEDTGSSSLPALMDSYISFDQTQAHADEFEQVPCFSIFSQNQANPIFNHMTTMEPKLPATTYGGAPNLGYCLDPLSCDRKVLKAVLSQITKMERNPLNQSLKGSTSFGEGSSESYLSEVGMPHMWNNY	present
R31	*glyceraldehyde-3-phosphate dehydrogenase*	880_3	38%	123	34,4/34,47	5,7/5,83	LVARVALQRNDVELVAINDPFITTDYMTYMFKYDTVHGQWKHFDVKVKDSKTLLFGEKAVAVFGTRNPEDIPWGEVGADYVVESTGVFTDKDKAAAHLKGGAKKVVISAPSKDAPMFVVGVNEKEYKPELDIVSNASCTTNCLAPLAKVINDRFGIVEGLMTTVHAITATQKTVDGPSSKDWRGGRAASFNIIPSSTGAAKAVGKVLPALNGKLTGMSFRVPTVDVSVVDLTVRIEKPATYEQIKAAIKEESEGKLKGILGFTDEDVVSTDFVGDSRSSIFDAKAGISLNENFVKLVSWYDNEWGYSTRVIDLIVHI	absent
R32	*ferritin*	1118_3	25%	80	28/28,30	5,96/5,64	MALAPSKVSPFSGFSLSDGVGAVRNPTCSVSLSFLNKKVGSRNLGVSASTVPLTGVIFEPFEEVKKEELAVPTAGQVSLARQYYADECESAINEQINVEYNASYVYHSLFAYFDRDNVALKGFARFFKESSEEEREHAEKLMKYQNTRGGRVVLHPIKNVPSEFEHVEKGDALYAMELALSLEKLVNEKLRSVHSVADRNKDPQLADFIESEFLSEQVEAIKKISEYVAQLRMVGKGHGVWHFDQSLLHDGHAA	present
R35	*protein disulfide isomerase-like protein*	131_3	26%	121	67/54,1	4,55/5,08	KEFVLTLDHTNFHDTVSKHDFIVIEFYAPWCGHCKKLAPEFEKAASILSSHDPPIVLAKVDANEEKNKELAMEYDVKGYPTIKIVRNGGKNIQEYKGPREADGIVDYLKKQNGPASTEIKSADEATALIGENKIAIVGVFPKFSGEEFDNFIALAEKLRAEYDFGHTLNAKHLPRGESSVAGPLIRLFKPFDELFVDSKDFHVDTLEKFVEESSTPVVTVFNNDPNNHPFVVKFFNSPNAKAMMFINFTAESAESFKSKYREAAEQYKQQGVSFLVGDVESSQGAFQYFGLKEEQVPLIIIQHNDGKKFFKSNLEADHIPTWLKAYKEGNIAPYVKSEPIPETNNEPVKVVVGENLQDIVFKSGKNVLLEFYAPWCGHCKQLAPILEEVAISYQSDANVIIAKLDATANDIPSDTFEVQGYPTLYFRSSSGTLSQYDGGRTKEDIIEFIEKNRDKPAQQEQDKPAHQEQVKDEQETGKDEL	absent
R36	*nucleoredoxin, putative*	5572_2	34%	123	52/37,35	6,95/4,72	DNTHDVVSLLSSPQRDFLLRNNGDQVKIESLKGKKLGVYFSASWCGPCRKFTPTLVEAYNEVVSKGDFEVVFASADEDEESFKGYFSKMPWLAIPFSDSETRSRLDELFHVRGIPHLVILEETGKVVTEDGVDIVREYGVDAYPFTSARIQELRAQEEEARRNQSVRSLLISPSRDFVISSDGNNILVSELEGKTVGLYFSLNSFQRSSEFTPKLVEVYEKLKAKGENFEVVLIPLDEDEESFKKVLESVPWLSLPFKDKFCGKLAQYFELSTLPTLVIIGPDGKTLNPNVAEAIEDHGVDAYPFTPEKFVELDEILKAREAAQTLESVLVS	present
R39	*ATPase catalytic subunit A*	gi|363806922	21%	105	66/68.9	6,04/5,29	MPAVYGARLTTFEDSEKESEYGYVRKVSGPVVVADGMAGAAMYELVRVGHDNLIGEIIRLEGDSATIQVYEETAGLMVNDPVLRTHKPLSVELGPGILGNIFDGIQRPLKTIAKRSGDVYIPRGVSVPALDKDTLWEFQPKKIGEGDLLTGGDLYATVFENSLMQHHIALPPDNMGKITYIAPPGQYSIKDTVLELEFQGVKKKFTMLQTWPVRTPRPVASKLAADTPLLTGQRVLDALFPSVLGGTCAIPGAFGCGKTVISQALSKYSNSDAVVYVGCGERGNEMAEVLMDFPQLTMTLPDGREESVMKRTTLVANTSNMPVAAREASIYTGITLAEYFRDMGYNVSMMADSTSRWAEALREISGRLAEMPADSGYPAYLAARLASFYERAGKVKCLGGPERTGSVTIVGAVSPPGGDFSDPVTSATLSIVQVFWGLDKKLAQRKHFPSVNWLISYSKYSTALETFYEQFDPDFINIRTKAREVLQREDDLNEIVQLVGKDALAEGDKITLETAKLLREDYLAQNAFTPYDKFCPFYKSVWMMRNIIHFYNLANQAVERGAGSDGQKISYTLIKHRMGDLFYRLVSQKFEDPAEGEAALVAKFQKLHEDLTNGFRNLEDETR	absent
R40	*PR1-like protein [Phaseolus vulgaris]*	gi|93359572	29%	117	16.3/16	4,3/4.83	TFEDQTTSSVAPATLYKAVAKDADTIFPKALPDSFKSVEIVEGNGGPGTIKKISFVEDGETKFVLHKIESIDEANLGYSYSIVGGVALPETAEKITFDSKLSDGPNGGSLIKLSITYHSKGDAPPNEDELKAGKAKSDSLFKAVEAYLLANP	absent
R41	*PR1-like protein [Phaseolus vulgaris]*	gi|93359572	65%	139	16/16	4.89/4.83	TFEDQTTSSVAPATLYKAVAKDADTIFPKALPDSFKSVEIVEGNGGPGTIKKISFVEDGETKFVLHKIESIDEANLGYSYSIVGGVALPETAEKITFDSKLSDGPNGGSLIKLSITYHSKGDAPPNEDELKAGKAKSDSLFKAVEAYLLANP	present

### Protein Extraction for 2DE Analysis

Protein sample preparations from leaves and roots were prepared according to methodology previously published [22], with minor modifications. One gram of each powdered tissue was homogenized in 25 mL 10% (m/v) TCA in acetone containing 0.007% (v/v) β-mercaptoetanol. The samples were then incubated at −20°C for 3 hours, centrifuged at 10,000 *g* for 30 minutes, the supernatant was discarded and two or three washes were performed. One wash step with 10% (w/v) TCA in water and two washes with cold acetone containing 2 mM PMSF (phenylmethylsulfonyl fluoride) were performed under the same centrifugation conditions as described above. The samples (TT, 42T, 42FS and 42RS from both tissues) were vacuum dried and solubilized using a solution containing 8 M urea, 2 M thiourea, 0.5% (w/v) CHAPS and 0.002% (v/v) IPG buffer. Protein content was estimated using the 2DE quant kit according the manufacturer’s instructions and bovine serum albumin as standard.

### 2DE Analysis

Precast 18 cm or 24 cm linear pH 3–10 IPG strips (GE Healthcare) were rehydrated overnight with 500 µg of protein preparations and De-streak reagent in a total volume of 350 µL for roots and 500 µL for leaf samples (GE Healthcare). Isoelectric focusing was carried out using an IPGphor III apparatus (GE healthcare) using the protocol: 500 V for 1 h, 1000 V for 1 h, 3000 V for 3 h and 80000 V to 70000 Vh. After isoelectric focusing, the IPG strips were rapidly removed and equilibrated for 15 min in 10 ml of solution A (50 mM Tris–HCl, pH 8.8; 6 M Urea; 30% glycerol; 1% SDS; 0.2% DTT and bromophenol blue dye), followed by another 15 min in 10 ml of solution B (50 mM Tris–HCl pH 8.8; 6 M Urea; 30% glycerol; 1% SDS; 3% iodoacetamide and bromophenol blue dye). After sequential equilibration in solution with 0.2% DTT and 2.5% iodoacetamide, the strips were transferred to an Ettan DALT 6 apparatus (GE healthcare). Electrophoresis experiments were carried out at 15°C for 1 h, with an initial current of 10 mA/gel, and then at 40 mA/gel until the dye front reached the bottom of the gel. The 2DE gels were stained using coomassie brilliant blue G-250. A total of three gels for each sample condition were run and analyzed.

### Image Analysis

The analytical gel images were obtained at a resolution of 600 dots per inch using the Image Scanner III (GE Healthcare), and the analysis performed using the software ImageMaster 2-D Platinum, Version 7.0 (GE Healthcare). Replicate gels for each treatment were used to create reference replicate groups. Spots were considered reproducible when they were well resolved in the three biological replicates. The measurement of each matched spot was carried out for each biological replicate, and the normalized volumes were computed using the total spot volume normalization procedure of the ImageMaster software. The experimental molecular masses (MW, kDa) of each protein were estimated by comparing them to the co- electrophoresed MW markers. The experimental p*I* of each protein was determined by its migration on IPG linear strips.

The abundance of each protein spot was estimated by the percentage of volume (% vol). Technical replicates of each biological sample were compared to each other by correlation analysis (R^2^) performed by the ImageMaster statistical package. Mean values ± SD of spot volumes from all the three biological replicates were used for further protein modulation comparison among the spot volumes of the other treatments. Spots presenting volume fold change were subjected to two- way ANOVA using the ImageMaster statistical package and a reference gel. Spots presenting volume fold change of more than 1.5 and *p* value<0.05, were considered to be differentially regulated. In addition, these spots were required to be consistently present in all the replicates. Down or up-regulated spots were excised from 2DE gels, trypsinized and identified by mass spectrometry.

### Protein Identification by Mass Spectrometry

All common spots compared in each condition from each biological replicate were manually excised from the 2DE reference gels and prepared for mass-spectrometric analysis.

Briefly, selected spots were washed three times with ultrapure water, three times with acetonitrile (50% v/v), and twice with 50 mM ammonium bicarbonate and 100% (v/v) acetonitrile. After the last wash with acetonitrile, the gel was carefully macerated with a sterile pestle. The acetonitrile excess was removed and the samples vacuum dried for 20 minutes. Then, the spots were rehydrated with 5–10 µL of trypsin solution (CaCl_2_ 2.5 mM in NH_4_HCO_3,_ 25 mM, containing 12.5 ng/mL of trypsin gold for mass spectrometry-Promega) for 45 minutes, on ice. The solution excess was removed and the spots covered with 5–8 µL of the same solution without enzyme. Then, the macerated gel spots were incubated at 37°C for 16 hours. The reactions were stopped by adding 1 µL of 1% (v/v) TFA to each digestion tube [23].

To obtain PMF spectra in an Autoflex II mass spectrometer, (Bruker Daltonics, Germany), 1 µL of each acidified digest product was applied in an AnchorChip™ plate (600 nm, Bruker) followed by 0.5 µL of dihydroxybenzoic acid (DHB) matrix (5 µg/µL in 30% CAN, and left to dry completely before analysis. The mass spectrometer was calibrated using Pepmix (Bruker Daltonics) and sample spectra containing auto-digested trypsin peptides were internally calibrated. Spectrometry was operated in the reflector mode for MS acquisition. Mass spectrometer parameters such as laser intensity and number of laser pulses per spectrum were manually adjusted for each sample to provide the best resolution and intensity.

The obtained MS spectra were analyzed *in house* using MASCOT 2.2 (Matrix Science, Oxford, UK) against an annotated protein sequence database from *P. vulgaris*, *P. coccineus* and *Glycine max* (EST database with 62,680 sequences) kindly provided by Dr. Marsolais [24]. Alternatively, searches were carried out against the NCBI non-redundant database (taxonomy of green plants). The following settings were used: enzyme, trypsin; MS tolerance of up to 0.3 Da; up to 1 missed cleavage; peptide charge of 1+; carbamidomethylation of Cys as fixed modification; oxidation of Met as variable modification. Only statistically significant protein identifications were accepted (p<0.05) followed by a stringent manual analysis of spectra to further avoid false-positives (matched peptides among the most intense peaks; non-random error distribution; presence of missed cleaved and cleaved peptides etc).

MASCOT analysis set the significance level at p<0.05, the obtained MS data indicated that the score obtained by peptide mass fingerprinting provided statistically most probable protein identification. The identification results were also evaluated by comparing the molecular mass and isoelectric point of the best scored hit with data observed in 2-DE gels. The spots were considered positive identifications if at least two specific peptides were identified in at least two of the replicates.

## Results and Discussion

The main goal of the present study was to analyze at the proteomic level the interaction between the mycoparasitic fungus *T. harzianum* (isolate ALL 42) and the host plant, *P. vulgaris,* in the presence or absence of the phytopathogenic fungi *F. solani* and *R. solani*. Trichoderma species have been described as promoters of host plant growth and as a trigger of plant defense response, however, there is still a lack of information concerning the molecular mechanism of these responses. Therefore, the present study may contribute to the better understanding of defense/growth promoting molecular mechanisms of these interactions, as well as, to discovering how the presence of *T. harzianum* can affect/modulate the interaction between phytopathogens and the host plant.

### Modulation of Plant Growth and Defense Response

The isolate ALL-42 was able to promote the growth of bean plants by increasing their overall size, foliar area, root area, and the number of secondary roots. It also showed ability to modify the root system architecture. A 1.14-fold size increase was obtained for plants treated with the mycoparasitic fungus (28.73 cm to 33.17 cm; p≤0.05). Increases were detected for foliar area, from 74.16 to 90.27 cm^2^, and for root area, from 391.19 to 613.86 cm^2^, respectively. Similar growth promotion effects were described for *T. harzianum*, *Trichoderma asperellum*, and *Trichoderma atroviride* in association with cucumber, maize, tobacco, *Pinus radiata* and tomato plants [14], [25]–[28]. However, the current work presents the first report of common bean growth promotion by *T. harzianum*. It should be noted that growth promotion is not a universal host plants response to association with *Trichoderma* species. Rubio and coworkers demonstrated a negative effect of *T. virens* (T-87) on the development of tomato plants. Its presence resulted in a decrease in root area and area/number of leaves [29]. This result is in agreement with previous studies which reported the relationship between host plant genetic background and their negative responses to the presence of *Trichoderma* species [30].

The ability of *Trichoderma* spp to promote plant growth was initially attributed to its antagonistic activity against harmful micro-organisms living in the rhizosphere or in the soil [31]. However, recently the growth promotion effects have been attributed to *Trichoderma*’s ability to colonize the root of the host plants enhancing their photosynthetic and respiratory rates [9]. *Trichoderma* spp colonize plant roots, invading the epidermis, and this association triggered plant metabolism by changing gene expression, and induced the production and secretion of hormones and growth factors that modulate plant growth [9], [26], [31]–[33]. The present data strongly suggest the potential use of *T. harzianum* from Brazilian Cerrado soil for the development of microbial inoculants for use in agricultural biotechnology and also as a biological model to understand the mechanisms of growth promotion of common bean plants triggered by this fungal specie.

Indeed, *T. harzianum* ALL-42 presented potential application in agriculture, since it was also described as an efficient inhibitor of *F. solani* and *R. solani* growth, either directly by antagonism or by the production of volatile metabolites [34]. As well as this, a rhizocompetent fungus was able to colonize and reduce the severity of white mold in *P. vulgaris* and to increase its number of pods in experiments carried out under field conditions [35].

**Figure 1:**
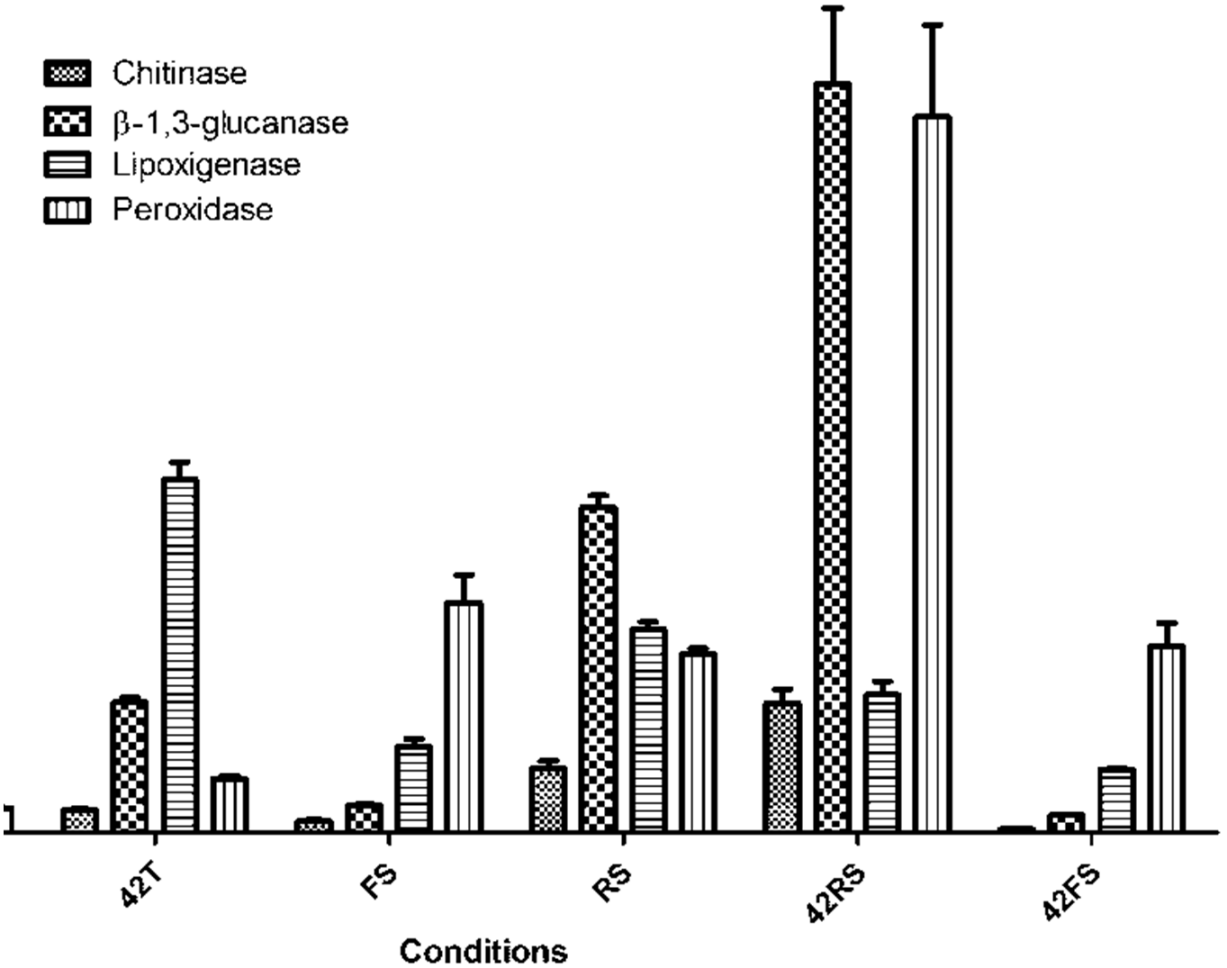
RT-qPCR analysis of defense-related genes expression in roots of common bean plants challenged and unchallenged with *T. harzianum* ALL 42, *F. solani* and *R. solani*. (TT) unchallenged plants; 42T plants challenged with *T. harzianum* ALL 42; FS plants challenged with *F. solani*; RS plants challenged with *R. solani*; 42FS double challenged plants *T. harzianum* ALL 42 and F. solani; 42RS double challenged plants *T. harzianum* ALL 42 and *R. solani.*

Common bean plants challenged by *T. harzianum* ALL-42 also presented differential expression pattern for defense response genes which encoded a chitinase (bch1), a β-1-3-glucanase (glu1), a lipoxygenase (lox) and a peroxidase (pod3) in comparison to unchallenged plants and those challenged by *F. solani* or *R. solani* alone. This response is in agreement with previous works which showed that this is a typical host plant response to its colonization by a symbiotic or pathogenic microorganism [9], [10], [18]. Plants challenged by *T. harzianum* ALL-42 presented up-regulation of Glu1, lox and pod 3 as compared with plants challenged by the phytopathogenic fungi. *T. harzianum* ALL-42 also seems also to potentiate common bean response to the presence of the phytopathogenic fungus *R. solani*, as shown by the increase in the levels of glu1 and pod3 for the double treatment in comparison to that obtained for plants in the presence of *R. solani* alone (Figure 1).

Therefore, *T. harzianum* ALL-42′s ability to act as a plant growth promoter correlates with its mycoparasitic activity and with its ability to associate to *P. vulgaris* roots, triggering expression values of genes encoding enzymes previously reported as playing a role in host plant defense response.

### 2DE Analysis

**Figure 2:**
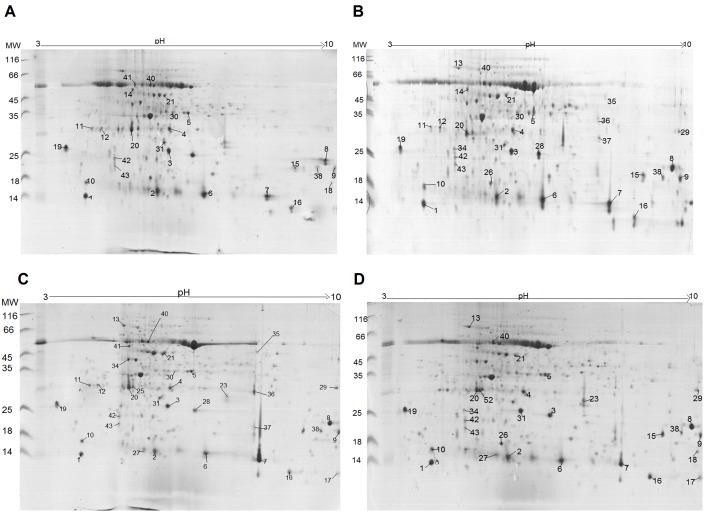
Reference proteomic maps from common bean leaves used in the statistical analyses to identify differentially expressed spots between unchallenged and challenged plants. (A) Leaf tissue of control plants cultivated in the absence of the Trichoderma isolate and phytopathogenic fungi; (B) Leaf tissue from plants cultivated in the presence of *T. harzianum*; (C) Leaf tissue from plants cultivated in the presence of *T. harzianum* and *F. solani* and (D) Leaf tissue from plants cultivated in the presence of *T. harzianum* and *R. solani*.

**Figure 3:**
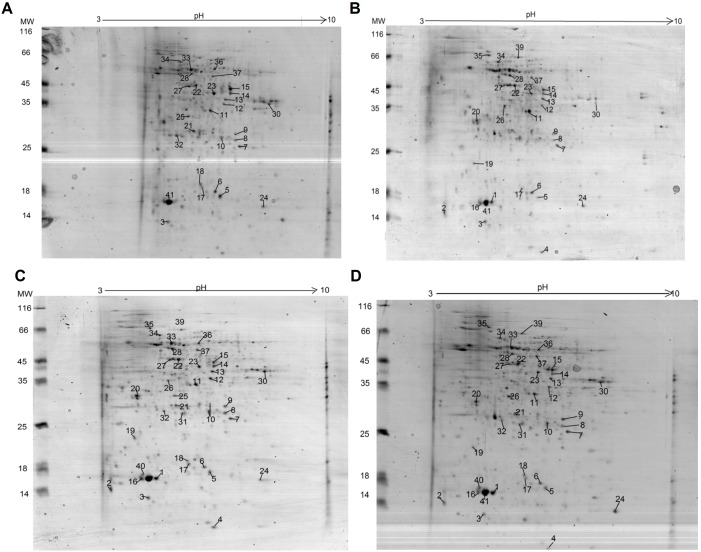
Reference proteomic maps from common bean roots used in the statistical of control plants cultivated in the absence of the *Trichoderma* isolate and phytopathogenic fungi; (B) Root tissue from plants cultivated in the presence of *T. harzianum*; (C) Root tissue from plants cultivated in the presence of *T. harzianum* and *F. solani* and (D) Root tissue from plants cultivated in the presence of *T. harzianum* and *R. solani*.

**Figure 4:**
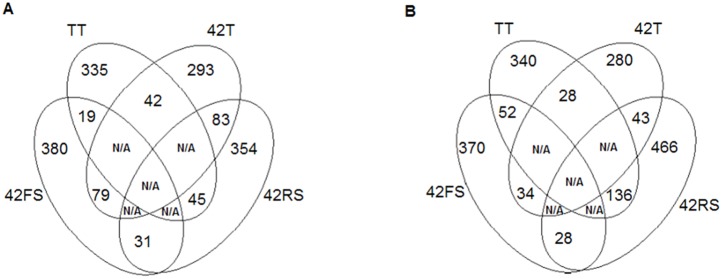
Four-way Venn diagrams comparing common spots with differential expression in TT, 42T, 42FS and 42RS conditions by ANOVA, considering p≤0.05 in (A) leaves and (B) roots of bean.

The proteomic mapping experiments were performed in technical and biological triplicates containing protein samples of leaves and roots from 10 common bean plants for each growth condition and biological replicate: untreated plants (TT), plants treated with the *T. harzianum* isolate (42T), plants double treated *T. harzianum* and *R. solani* (42RS), plants double treated *T. harzianum* and *F. solani* (42FS). The technical and biological replicate maps for each growth condition were analyzed using the ImageMaster software, and reference gels were constructed. Each replicate image was compared to its reference gel and correlation indexes between the values of 0.97 and 0.99 were obtained, showing the technical reproducibility. Bean leaf maps showed protein spots with molecular masses ranging from 14 to 60 kDa (Figure 2), while the root maps showed spots in a range of 10 to 70 kDa (Figure 3). The proteomic maps are quite different from each other; spots up or down-regulated and exclusively detected in some maps were observed. The largest number of spots was detected for untreated and double treated common bean plants (*Trichoderma*-phytopathogenic fungus with *R. solani* or *F. solani*) in comparison to *T. harzianum*-treated plants (Figure 4).

The normalized spots volumes (V (%) = volume of each spot/total volume of all protein spots on the same gel) were analyzed in an ANOVA test (p<0.05) using the data from each of the three biological replicate maps from control, *Trichoderma*-inoculated plants and *Trichoderma*/phytopathogenic fungi-inoculated plants. The differential spots were those presenting *p-value* above 0.05 and normalized ratio value equal to or larger than 1.0 (up-regulated), and equal to or lower than 0.35 (down-regulated) in comparison to the relative volume values obtained for the untreated plants map. Exclusive spots were detected for each growth condition in comparison to the control treatment (untreated plants); for leaves were observed 42, 19 and 45 exclusive spots for the treatments 42T, 42FS and 42RS, respectively.

**Figure 5:**
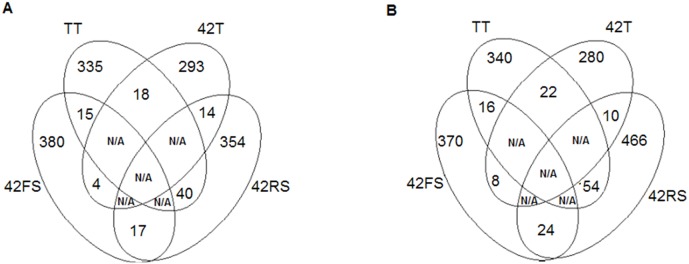
Four-way Venn diagrams comparing exclusive spots in TT, 42T, 42FS and 42RS conditions by ANOVA, considering p≤0.05 in (A) leaves and (B) roots of bean.

The comparison of root maps identified 28, 52 and 136 exclusive spots for the treatments 42T, 42FS and 42RS, respectively (Figure 5). Therefore, the fungal presence significantly modulated the spot pattern and distribution for common bean plants challenged or not by the presence of the fungi. This alteration was remarkable for the leaf map of plants challenged with the *Trichoderma* isolate, suggesting its ability to induce a systemic response. This is in contrast to that observed for phytopathogenic fungus- treated plants, which showed a strong local response against their presence.

Common spots presenting increased or decreased relative volumes were also detected for each growth condition in comparison to the control treatment (unchallenged plants). For leaves were observed 18, 15 and 40 common spots for the 42T, 42FS and 42RS treatments, respectively. The root maps presented 22, 16 and 54 common spots with differential relative volumes for the 42T, 42FS and 42RS treatments, respectively (Figure 4). Comparisons between the proteomics maps obtained for common bean tissues of challenged plants were also carried out. For leaves 14 differential spots were obtained for comparison between the treatments 42T and 42RS, 4 for 42T and 42FS, and 17 for 42FS and 42RS. For the root maps 10 differential spots were obtained on comparison between the treatments 42T and 42RS, 8 for 42T and 42FS, 24 for 42FS and 42RS. Therefore, the physiological change triggered in common bean plants by the presence of the fungi seems to involve two main mechanisms: presence or absence of proteins and up/down regulation of the production of some proteins. The spot occurrence and pattern of distribution were also dependent on the kind of fungus interacting with the host plant.

### Protein Identification

After determination of the relative volumes for each protein spot, defined by normalized volume, a parallel comparative analysis was conducted between untreated plant maps and the maps for the inoculated conditions, to characterize protein spots with differential occurrence, distribution and % volume. Initially, a total of 299 proteins spots were identified with differential % of volume values for roots and 321 for leaves, based in their normalized volumes. After parallel comparative analysis, only those spots showing consistent results in all parallel gels were selected. The differential spots from leaf and root maps were excised from gels and submitted to mass spectrometry analysis using the Autoflex II equipament, (Bruker Daltonics, Germany), for identification.

A total of 48 protein spots were identified; 19 for leaf maps (Table 1) and 29 for root maps (Table 2). Several proteins remained unidentified despite the good quality of the peptide spectrum and the use of an annotated protein sequence database containing *Phaseolus vulgaris*, *P. coccineus* and *Glycine max* protein sequences. The scarcity of protein sequence entries in the available databases limits the identification of proteins and has been considered as a bottleneck to protein mining using proteomic approaches [36].

**Figure 6:**
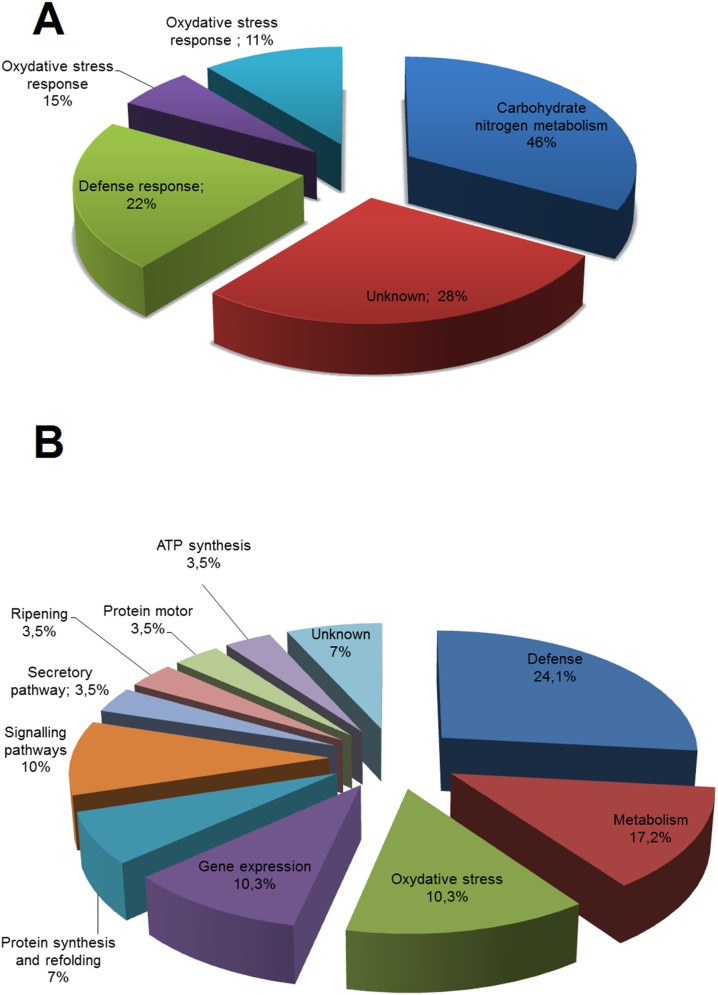
Functional categorization of *P. vulgaris* identified proteins. (A) Leaf tissue and (B) Root tissue.

The leaf-identified proteins were categorized into five functional categories: metabolism (33%), defense-related (22%), unknown (28%), oxidative stress response (11%) and protein synthesis (6%) (Figure 6A). For root-identified proteins a different pattern of distribution was observed, with eleven functional categories (Figure 6B), the major represented classes being defense (24%), metabolism (17.2%) and oxidative stress response (10.3%) as were observed for leaf-identified proteins. In addition, signaling pathway (10%) and gene expression (10.3%) proteins were also frequently represented (Figure 6B).

### Identified Proteins in Leaf Tissues

**Figure 7:**
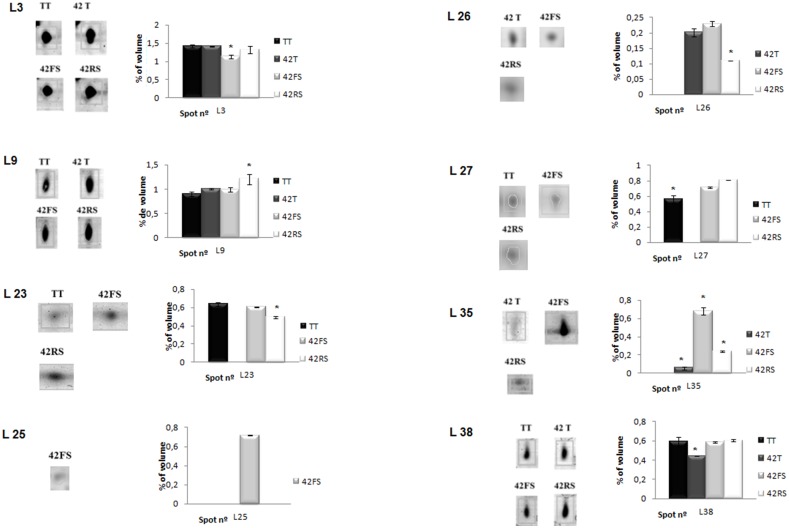
Differential distribution pattern of leaf- identified proteins in the four conditions described: TT, 42T, 42FS and 42RS.

Spots identified as Rubisco (Small subunit), Cinamoyl CoA reductase (CCR) and Aminomethyltransferase (L7, L26 and L35, respectively) (Figure 7) were increased in the leaves of *Trichoderma*-challenged common bean plants. Up-regulation of rubisco might result in an increase in photosynthesis rates, as previously suggested for the interaction between *T. harzianum* T22 or *T. virens* and maize [14], [37]. Maize shoots from *T. harzianum* T22 treated plants presented up-regulation of photosynthesis-related genes including two forms of rubisco large subunits. *T. virens* is able to modulate photosynthesis-related genes expression in maize leaves as shown by up-regulation of mRNA levels of a rubisco small subunit and an oxygen-evolving enhancer.

In both cases, up-regulation of these genes was accomplished by growth promotion and increase in leaf greenness. This type of response was also described for *Trichoderma* species in association with cucumber, maize and tomato plants [26], [10], [14], [28]. Therefore, it is widely accepted that growth promotion is mediated by an increase in photosynthetic rates.

The cinamoyl CoA reductase, CCR (spot L26, Figure 7), also up-regulated in leaves of 42T-challenged plants catalyses a key reaction in the phenylpropanoid pathway, converting cinnamoyl - CoA esters to monolignols, which present antimicrobial activity [38] and can also be polymerized to form the complex polymer lignin. This result suggests that the *Trichoderma* isolate could increase the lignification rate and antimicrobial compound production protecting the host plant against a subsequent pathogen attack and inhibiting growth and host tissue invasion. The CCR role in defense response has been described in different pathosystems such as interactions between Arabidopsis and *Xanthomonas campestris pv. campestris* and between rice and *Xanthomonas oryzae* pv. *oryzae*, where up-regulation of CCR-encoding genes was seen in the leaves of pathogen-infected plants [39], [40].

One spot identified as a Glutathione S-transferase GST 13, (Spot L25, Figure 7), was up-regulated only in the presence of the phytopathogenic fungus *F. solani*, while a GTP-binding protein and uncoupling protein 1a (spots L38 and, L27, Figure 7) were up-folded in the presence of both of the phytopathogenic fungi (*R. solani* and *F. solani*). The two former proteins have been described as important in minimizing the effects of reactive oxygen species (ROS) produced as a typical defense response triggered by pathogen recognition at the infection site [41]–[44]. The increase in GST levels was also described in other pathosystems such as Arabidopsis and *Alternaria brassicicola* using proteomic and transcriptomic approaches and has been considered as an early marker of defense response in Arabidopsis [45], [46].

### Identified Proteins in Roots Tissues

**Figure 8:**
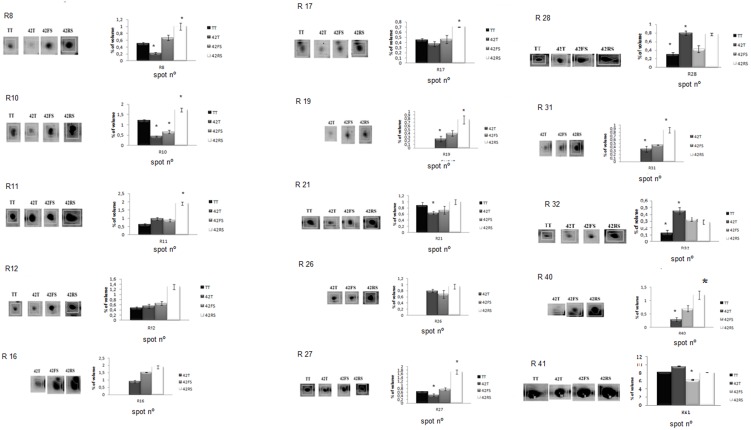
Differential distribution pattern of root- identified proteins in the four conditions described: TT, 42T, 42FS and 42RS.

The spots identified as defense-related proteins (spots R10, R11, R12, R19 R36, R40 and R41, Figure 8) presented differential relative volumes between the challenged and unchallenged plants. The spots R10, R11, R12, R19 and R40 identified as a chalcone isomerase, Pto-like kinase SG5-3b, isoflavone reductase, acyl-CoA-binding protein and PR1-like protein, respectively, were remarkably up-regulated in the root map of common bean plants challenged with *R. solani* in comparison to the maps for *F. solani*-challenged plants (Figure 8). The different relative volumes of these spots in the maps of plants challenged with the phytopathogenic fungi suggested that the host plant response could be pathogen dependent [47], [48]. The three former proteins are defense proteins recognized as pertaining to the HR response and play a role in the incompatible plant-pathogen interactions commonly described after the contact between pathogen and host plant [47], [48].

The spots identified as PR1-like protein (R40) and acyl-CoA-binding protein (R19) were also up-regulated in the root maps of common bean plants challenged with the *Trichoderma* isolate (Figure 8), but presented lower relative volume values than those detected in the presence of the phytopathogenic fungi. The role of acyl-CoA-binding protein in defense response of plants was previously described for Arabidopsis transgenic mutants over-expressing the *ACBP3-*encoding gene. These mutants showed constitutive expression of pathogenesis-related genes (*PR1*, *PR2*, and *PR5*), cell death, and hydrogen peroxide accumulation in leaves leading to an enhanced resistance to the bacterial pathogen *P. syringae* DC3000 [49]–[51].

This might suggest that the *T. harziamum* isolate is able to induce a basal defense response possibly priming the host plant to respond to the pathogen attack through a stronger defense reaction once infection has occurred. This is the most common response thus far described in *Trichoderma*/host plant (Cucumber, cotton and maize) interaction studies [14].

Indeed, the up-regulation of isoflavone reductase and acyl-CoA-binding protein was observed also in avocado and Arabidopsis plants after infection with *Phytophthora cinnamomi* and *Pseudomonas syringe* pv. tomato DC3000, respectively [46]–[50]. Chalcone isomerase and isoflavone reductase (CHS) are key enzymes in the synthesis of isoflavonoid phytoalexins, which play important roles in plant defense, with protective activity against micro-organisms and herbivores and acting as a mediator of the salicylic acid defense pathway [52], [53]. In addition, it was also observed for soybean plants that isoflavone levels increase significantly after infection by the phytopathogenic fungus *Sclerotinia sclerotiorum* [54].

Four spots identified as isoforms of glutathione S-transferase (R8 and R220), peroxiredoxin (R17) and monodehydroascorbate reductase (R28) grouped as oxidative stress response proteins were up-regulated in the root map of common bean plants challenged with *R. solani* (Figure 8). This sort of result was also described for the leaf proteomic map of common bean plants challenged with this phytopathogenic fungus. As discussed above, oxidative stress response proteins played a crucial role in infected plants to minimize the effects of the reactive oxygen species (ROS) produced as a typical defense response triggered by pathogen recognition at the infection site [41]–[43]. Arabidopsis plants after the infection by an avirulent strain of *Pseudomonas syringae* also showed the ability to increase the early expression of two glutathione S-transferase genes, *AtGSTF2* and *AtGSTF6*. Their expression correlated with the production of ethylene and SA accumulation after the pathogen attack, and both genes are SA- and ethylene-inducible [55].

Typically, levels of reactive oxygen species are low in plant cells, but increase during infection by microorganisms. In our results, the greatest changes in the bean proteome occurred in the presence of *R. solani*, suggesting that this pathogen triggers more severe symptoms when compared to *F. solani*.

The spot R21, identified as a member of the 14-3-3-like protein family and classified as a signaling pathway protein, was up-regulated in proteomic maps of common bean plants challenged with the phypathogenic fungi and with *Trichoderma* isolate (Figure 8). The role of one 14-3-3-like protein isoform (TFT7) in controlling MAPK signaling pathways was described for tobacco and tomato plants, and this activity is related to resistance against *P. syringae* and hypersensitive response-induced cell death [56], [57]. Whereas that MAPK can act as a regulator of cell-death signaling networks, resulting in lesion formation around the infection site occasioned by programmed cell death and, in turn preventing the pathogen propagation to the other plant tissues, it can be supposed that the up-regulation of spot R21 can be can be intrinsically related to the plant immunity.

Proteins related to the control of gene expression and protein folding were also up-folded in the root proteomics maps of common bean plants challenged by the *Trichoderma* isolate and the phytopathogenic fungi (Figure 8). These include a histone acetyltransferase complex component (spot R16) and NAC1 domain protein (spot R26). The transcription factors classified as NAC family members presented a crucial role in a wide range of plant developmental processes, such as shoot lateral root development and defense responses [58]–[60]. The role in defense response was described for two NAC family proteins, ANAC019 and ANAC055, acting as activators of JA-induced defense genes downstream of MYC2 [61]–[63].

Metabolism proteins were also identified in the root proteomics maps, up-regulated for common bean plants challenged with the phytopathogenic fungi and *T. harzianum* isolate. The spots, R27, R31 and R32, were identified as fructokinase, glyceraldehyde-3-phosphate dehydrogenase (GAPDH) and ferritin (Figure 8). These proteins play a crucial role in carbohydrate metabolism, as well as in iron mobilization and transport in aerobic metabolism, respectively. Ferritin was highly expressed in the proteomics maps of *Trichoderma*-challenged plants, suggesting an important role of this fungus in increasing the plant’s ability to mobilize and transport iron.

### Validation of Differential Protein Accumulation

**Figure 9:**
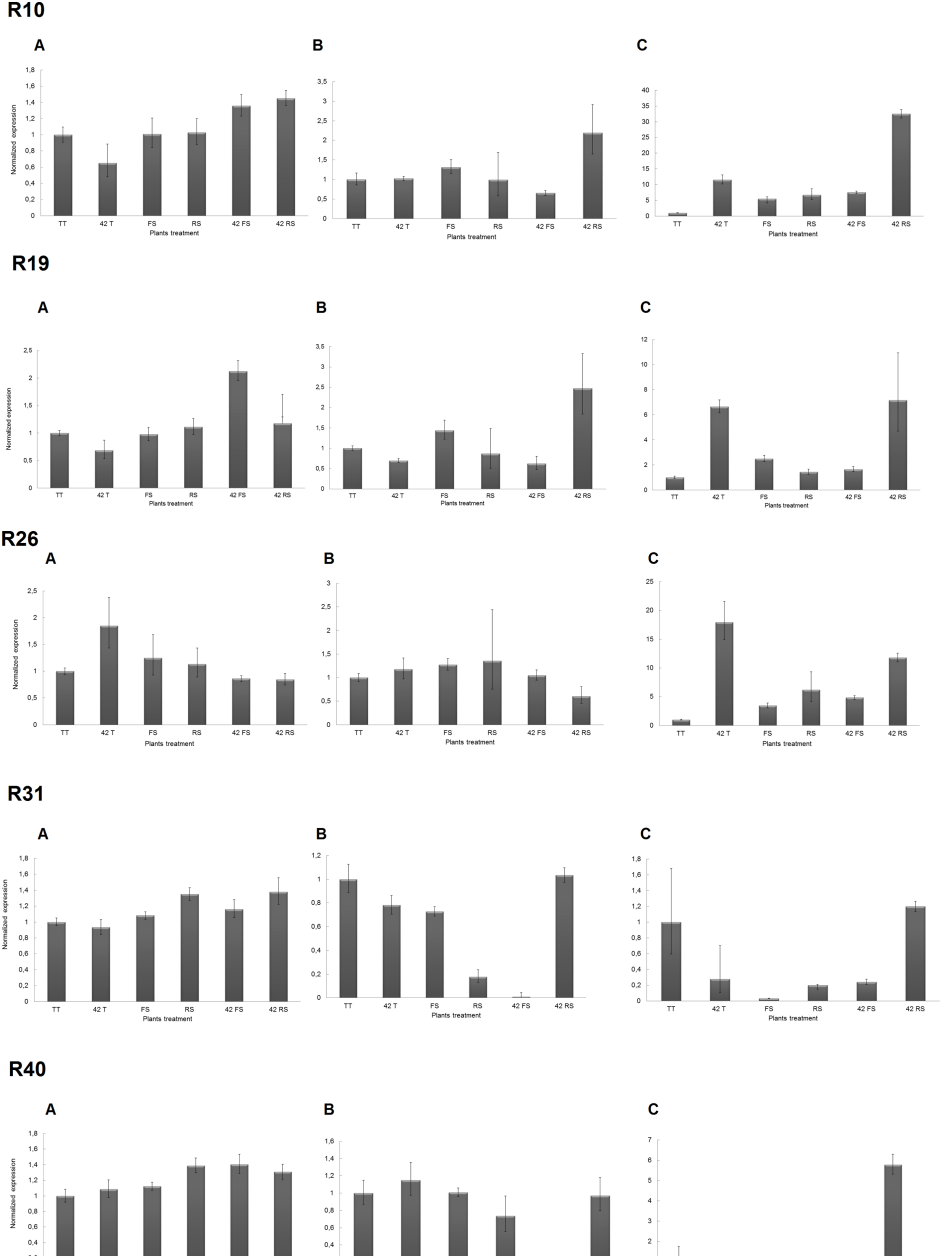
RT-qPCR analysis of Chalcone isomerase (Chal), acyl-coA-binding protein (Ac), NAC1 domain protein (Nac), glyceraldehyde-3-phosphate dehydrogenase (Ghd) and PR1-like protein (Pr1)-enconding genes in roots of common bean plants challenged and unchallenged with *T. harzianum* ALL 42, *F. solani* and *R. solani*. (TT) unchallenged plants; 42T plants challenged with *T. harzianum* ALL 42; FS plants challenged with *F. solani*; RS plants challenged with *R. solani*; 42FS double challenged plants *T. harzianum* ALL 42 and F. solani; 42RS double challenged plants *T. harzianum* ALL 42 and *R. solani.* Time of growth: (A) 7, (B) 14 and (C) 21 days.

The genes encoding chalcone isomerase, acyl-coA-binding protein, glyceraldehyde-3-phosphate dehydrogenase and PR1-like protein presented different expression patterns between the challenged and unchallenged plants in agreement with results described above for the proteomics maps (Figures 8 and 9). These transcripts were remarkably up-regulated in common bean plant roots challenged with *R. solani* in comparison with the other treatments (Figure 9). Substantial transcript level increases were mainly detected after 14 and 21 days of growth suggesting a late plant response to *R. solani* infection (Figure 9).

*T. harzianum* ALL-42 was able to modulate the transcript levels of acyl-coA-binding protein and PR1-like protein-encoding genes with expression values lower than those detected in the presence of *R. solani* (Figure 9). This result is in agreement with the proteomic data (Figures 8 and 9). Correlation between proteomic data and transcript level was also confirmed for NAC1 domain protein-encoding gene (Figures 8 and 9). NAC1 transcript levels notably increase for common bean plants challenged with the phytopathogenic fungi and *T. harzianum* isolate after 21 days of growth (Figure 9). Therefore, RT-qPCR expression profiles were in complete agreement with the proteomic data.

As suggested above for defense response gene expression, *T. harzianum* ALL-42 seems to potentiate the expression levels of the evaluated genes, as shown by the increase in the levels for the double treatment in comparison to that obtained for plants in the presence of *R. solani* alone (Figure 9).

## Conclusions

In summary, the presence of the *T. harzianum* isolate ALL-42 modulates the common bean plant’s metabolism and triggers its defense response. Furthermore, the isolate ALL-42 acts as a primer to trigger the host defense response, which is potentiated after invasion by the phytopathogenic fungi in a local and systemic response. However, we are unable to make a suggestion as to which is the main plant defense mechanism triggered by *T. harzianum* ALL-42, as we identified proteins with key roles in the different plant defense responses that have been previously described (SAR, ISR and HR).

These responses were also described for *T. harzianum*, *T. virens* and *T. asperellum* in association with tomato, cucumber, potato, cotton and *A. thaliana* [61]. They have been described as inducers of systemic induced resistance against a set of pathogenic fungi and bacteria for different host plants, acting by increasing the JA/E- responsive gene expression levels [64]. Moreover, they are also related to the triggering of SAR response leading to an increase in the SA-inducible gene expression levels, and acting as long-lasting up-regulators of the salicylic acid pathway even in the absence of a pathogen [30], [65].

In the present work, a set of proteins up-regulated during the interaction with *T*. *harzianum* and pathogenic fungi were identified and will contribute to a better understanding of the molecular basis of the common bean-*T. harzianum* dialogue in the presence/absence of *R. solani* or *F. solani* in complementary studies. Moreover, this work also contributed to an increase in number of proteins cataloged as presenting a key role in fungal pathogenesis and symbiosis using common bean as a biological model.
